# P2X7 receptor antagonist delivery vehicle based on photocrosslinked amphiphilic hybrid gels†

**DOI:** 10.1039/c8ra01460d

**Published:** 2018-05-18

**Authors:** Derya Aydin, Seda Kizilel

**Affiliations:** Department of Chemical and Biological Engineering, Koc University Sariyer Istanbul Turkey 34450 skizilel@ku.edu.tr +90-212-338-1548

## Abstract

We report here a method for the synthesis of a unique hybrid gel system for the sustained delivery of P2X7 receptor (P2X7R) antagonist. P2X7R has been reported as a key mediator in inflammatory processes and controlled delivery of this molecule would be critical for the treatment of inflammatory arthritis. The hybrid gel designed here for the sustained delivery of P2X7R antagonists is based on crosslinked hydrophobic styrene-butadiene-styrene (SBS) polymer as a continuous network, where hydrogel particles prepared with hydrophilic poly(ethylene glycol) (PEG) were embedded into this system. PEG hydrogel particle-incorporated SBS gels were characterized through electron microscopy, water contact angle observations, and strong mechanical properties were confirmed through nanoindentation measurements. The release of P2X7R antagonist from these hybrid hydrogel-elastomer system demonstrated a sustained drug release profile up to 28 days at physiological pH, which was not observed in earlier reports. We obtained drug release percentages ranging from 49.72% to 93.04% which indicated the tunability of release through SBS crosslinking and hydrophilic/hydrophobic nature of SBS. This tunability is significant to achieve simultaneous improvements in drug efficacy with reduced side effects. CellTiter-Glo luminescence measurements using human kidney cells revealed that these networks are non-toxic and highly biocompatible with percent cell viabilities of higher than 85%. The approach presented here with crosslinked, amphiphilic and elastic SBS gel systems is not only promising for extended release of P2X7R antagonist but could also allow for incorporation of different molecules so that simultaneous/sequential and extended release profiles for therapeutic molecules could be achieved.

## Abstract 2

We report here a method for the synthesis of a unique hybrid gel system for the sustained delivery of P2X7 receptor (P2X7R) antagonist.

## Graphical abstract

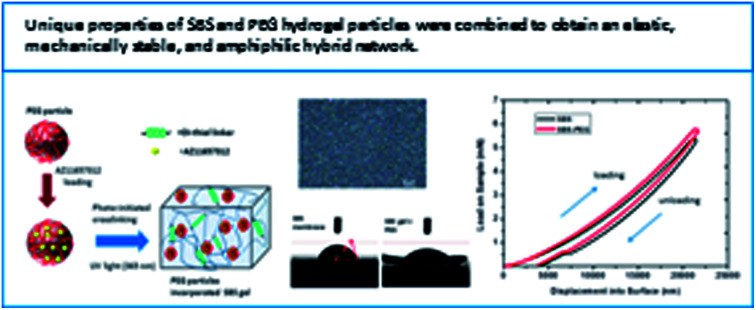

## 1. Introduction

SBS is commonly used as a rubber substitute due to its desirable properties such as high tensile strength and elasticity properties.^1,2^ It is a synthetic triblock copolymer composed of long chains of polybutadiene and polystyrene. Polystyrene is tough and this contributes to the durability of SBS, while polybutadiene brings rubber-like properties to SBS. SBS has been used in a variety of industries where durability is important, such as building materials (as an additive to bitumen to improve mechanical properties of roads), shoes, tire treads, biomedical devices and other places. However, the potential of SBS for biomedical applications has only been considered in limited studies.^3–5^

SBS can be crosslinked to create slightly hydrophobic networks through thiol–ene photopolymerization.^6–8^ Crosslinked SBS can be synthesized through the addition of radicalized thiol groups onto double bonds present in poly(butadiene blocks). PEG is a well-known biocompatible polymer that can be crosslinked *via* photopolymerization to obtain a hydrophilic gel network which enables pharmaceutical loading.^9^ We and others have used eosin Y and triethanolamine (TEA) photoinitiator system in free radical photopolymerization of poly(ethylene glycol) (PEG) hydrogels to carry out the crosslinking reaction under visible (wavelength ∼500 nm) or UV light conditions.^10–12^ In our previous study, we designed covalently crosslinked SBS network through UV photoinitiated thiol–ene reactions using dithiothreitol (DTT) as a crosslinker and eosin Y-TEA photoinitiation system to initiate crosslinking reactions with TEA radicals.^3^

Extended drug release is important for efficient treatment of various diseases such as periodontitis and severe bacterial infections since these physiological processes require repeated administration of drugs.^13,14^ Sustained release of drugs from a network depends on hydrophilic/hydrophobic nature of the drug and the carrier matrix, network properties of matrix and degradability of its components.^13^ Sustained release of drugs from various materials such as hybrid polymers, composites and hydrogels in the form of bulk material or nanocarriers have been reported.^15–22^ For example, two-component system from adamantane-modified and cyclodextrin modified hyaluronic acid were designed to prepare injectable hydrogels for sustained release of small molecules.^18^ In another report, vincristine, hydrophobic and commonly used chemotherapeutic agent, was encapsulated within a peptide hydrogel and vincristine was shown to release constantly over the course of one month.^19^

P2X7R antagonists represent a novel approach for the treatment of inflammatory arthritis, a long-term disease that causes inflammation and deformity of the joints, and affects about 1% of the world population. P2X7R activation is critical in inflammatory processes and repeated injections are required to achieve long lasting and constant patient relief.^23–25^ Hence, long-acting formulations that act by single-dose injection of anti-inflammatory drugs for P2X7R activation would be clinically desirable.^26^ Therefore, slow release of a potent anti-inflammatory drug, P2X7R antagonist, presents a novel approach for the treatment of inflammatory arthritis.

Elastomers represent other class of materials which have been considered in wound dressing and drug delivery applications.^19,27–32^ For example, sustained release of berberine and chlorhexidine from biodegradable elastomer poly(glycerol-sebacate) (PGS) were monitored up to 60 days and drug loaded PGS groups demonstrated antibacterial properties against periodontal disease pathogens.^29^ In another study, a silicone elastomer, porous PDMS demonstrated abundant void space for the preloading of pharmaceuticals and promoted burst release (about 80% delivery achieved in 30 min), with improved permeability of water vapor.^27^ In another report, slow release of NO was achieved from photo-crosslinked biodegradable poly(diol citrate) elastomeric network.^30^ Delivery of non-steroid anti-inflammatory drugs from a micropillar PDMS patch was also investigated and continuous release of drug was monitored for one week.^31^ However, the release of a drug from a hydrophilic hydrogel-rubber elastomer hybrid system was not developed in previous reports.

Among different variety of elastomers, SBS was considered in limited biomedical applications where hydrophilic compounds commonly were grafted onto SBS to increase biocompatibility of the resulting copolymer.^4,33^ For example, Yang *et al.* developed graft polymerization of SBS with hydrogels such as hydroxyethyl methacrylate (HEMA) and amino ethyl methacrylate (DMAEMA) to overcome limitations associated with poor mechanical properties of hydrogels upon swelling.^5^ They further added heparin to the grafted copolymer and observed high water content of copolymer and low water contact angles with increased amount of grafting and heparin. They also observed superior mechanical properties of copolymers compared to that of hydrogel alone. In another study, *n*-isopropylacrylamide (NIPAAm) was grafted onto SBS *via* solution polymerization to improve the water absorption and thermo sensitivity of SBS.^33^ Design of a hybrid gel system prepared with photo-crosslinked SBS network that includes embedded PEG hydrogel particles and release of P2X7R antagonist from this type of rubber elastomer-hydrogel system were not considered previously.

**Scheme 1:**
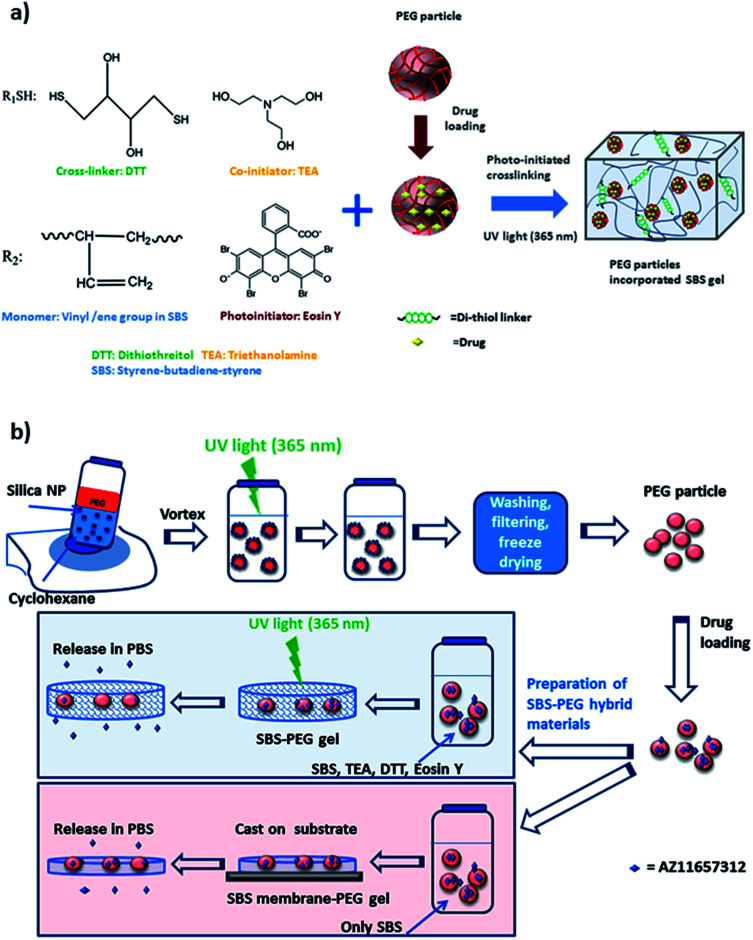
(a) Components of amphiphilic and crosslinked SBS-PEG gel network, (b) summary of preparation steps for the synthesis of SBS-PEG and SBS membrane-PEG.

In this study, we utilized hydrophilic character of PEG hydrogel particles within SBS to achieve slow and sustained drug delivery, where we obtained high mechanical strength and hydrophobicity through SBS for the release of an amphiphilic drug, P2X7R antagonists through a novel hybrid gel network. We prepared two different types of hybrid networks: SBS-PEG hybrid gels and SBS membrane-PEG gels (Scheme 1). SBS-PEG hybrid gels were prepared through suspension of crosslinked PEG particles in SBS solution followed by crosslinking of SBS solution through photopolymerization. SBS membrane-PEG gels were prepared by incorporation of PEG particles into SBS solution followed by casting of the PEG particle incorporated SBS polymer solution onto glass substrate surfaces. The main advantages of the system developed here are: (i) ability to crosslink hydrophobic SBS network; (ii) incorporation of hydrophilic PEG hydrogel particles; (iii) casting hydrophobic SBS membrane bearing hydrophilic particle islands onto surfaces. Finally, the preparation of these amphiphilic hybrid networks in the form of a crosslinked network or templated structure promoted sustained delivery of P2X7R antagonist, which is significant for a variety of biomedical conditions including the treatment of inflammatory arthritis.

## 2. Materials and methods

### 2.1. Materials

**Fig. 1:**
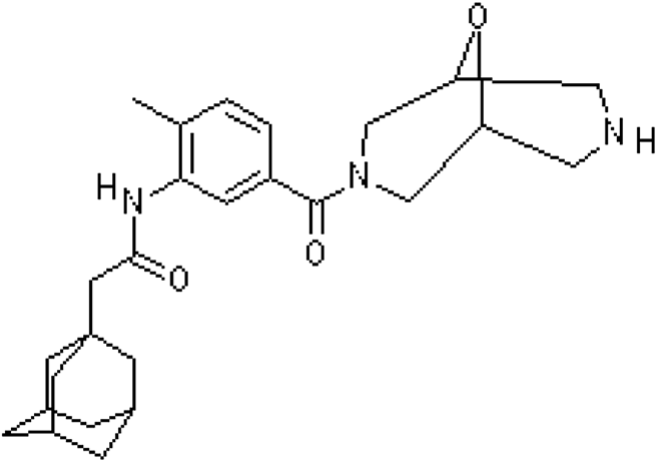
Structure of P2X7R antagonist.

SBS D1101 (S : B wt fraction 70 : 30) was purchased from Kraton; dl-dithiothreitol (DTT); eosin Y and ethanol (99.8%) were purchased from Sigma-Aldrich; triethanolamine (TEA) and cyclohexanone (99.9%) were purchased from Merck (Darmstadt, Germany) and phosphate-buffered saline (PBS) tablets were obtained from Amresco (Solon, Ohio). Silica nanoparticles (AEROSIL 816) were used for the preparation of emulsions. PEG diacrylate (PEGDA) (575 Da) was purchased from Laysan Bio. Vinyl-2-pyrrolidinone (VP) was purchased from Aldrich. AZ11657312 (P2X7R antagonist) was kindly provided from AstraZeneca (Istanbul, Turkey) (Fig. 1). Dulbecco modified Eagle medium (DMEM) was purchased from Gibco Life Tech. Penicillin/streptomycin, l-glutamine and FBS was purchased from Sigma-Aldrich.

### 2.2. Synthesis of PEG particles and encapsulation of particles in crosslinked SBS

Prepolymer solution was prepared with a concentration of 30 (w/v) PEGDA, 225 mM TEA and 37 mM VP in 10 mM PBS at pH 8. EosinY was added to the prepolymer solution at a final concentration of 0.025 mM. Stock nanoparticle solution with a concentration of 1.5% (w/v) was prepared. Prepolymer solution (with an internal volume fraction of 25% (v/v)) is stabilized by silica nanoparticle dispersion (1.5 wt/v%) in cyclohexanone to obtain Pickering emulsion which we developed and used in our previous studies.^34,35^ The particle dispersion was kept at room temperature. PEGDA prepolymer (0.5 mL) was added to 1.5 mL of nanoparticle stock solution under high shear by applying vortex. As prepolymer solution was mixed in cyclohexanone, the aqueous solution formed well-dispersed droplets which were stabilized by silica nanoparticles. Synthesis of PEG particles was monitored using a TA Instruments AR/DHR series rheometer (New Castle, DE) with a UV light attachment. Nanoparticle stabilized prepolymer emulsion (600 μL) was illuminated with UV light at 365 nm, with a flux of 100 mW cm^−2^ for 900 s. Next, PEG-cyclohexane emulsion was washed with ethanol and distilled water, filtered and dried to obtain dry PEG particles. PEG hydrogel particles were incubated in drug solution for the loading of drug. A prepolymer solution containing 7.5 wt% SBS, 2.5 wt% DTT, 0.075 mM eosin Y, and 225 mM TEA was prepared in cyclohexanone. PEG particles loaded with drug were dried and dispersed in cyclohexanone at 10 mg mL^−1^ concentration. PEG particle dispersion was added to SBS prepolymer solution with 0.16 (v/v) fraction. The mixture was then crosslinked with the UV light of the rheometer at 365 nm, with a flux of 150 mW cm^−2^ under 1% strain, at a frequency of 10 Hz for 3000 s. The crosslinked SBS networks were obtained as disks over a quartz sensor surface covered with a rotating plate with a diameter of 2 cm, and a fixed gap size of 1000 μm. Schematic representation and components for the preparation of amphiphilic and crosslinked SBS with embedded PEG hydrogel particles are demonstrated in Scheme 1.

### 2.3. Morphological characterization of emulsions, PEG particles and SBS-PEG gels

Emulsions were observed with electron and light microscope. Scanning electron microscope (SEM) operated between 1.00 and 3.00 kV was used to characterize structure and morphology of PEG particles and SBS-PEG gels. PEG particles were also characterized by Fourier Transform Infrared Spectroscopy (FTIR) spectroscopy to confirm gelation and crosslinking of PEG particles. Static water contact angle measurements were performed on a Dataphysics OCA 35 instrument at room temperature (24 ± 2 °C) to investigate the wettability of surfaces. Triple distilled water (10 mL deionized) was used for contact angle measurements.

### 2.4. Nanoindentation tests

Depth sensing nanoindentation test (Agilent G200, USA) with Berkovich diamond indenter tip was used to characterize the mechanical properties of SBS and SBS-PEG gels. During nanoindentation, the test tip penetrates into the sample surface with an indentation load of *P* (N) and indentation depth of *h* (m). Each indentation consists of loading and unloading phase. The hardness and elastic modulus of samples were obtained from the instrument calculating from load–displacement curves by using Oliver–Pharr method.^36,37^ The hardness of the material is obtained from loading phase and defined as:1
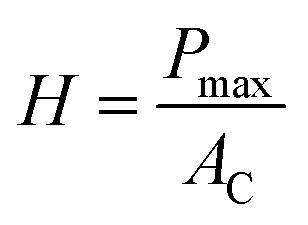
where, *P*_max_ is the applied maximum load and *A*_C_ is the horizontal projection of the contact area of tip at the end of loading phase. In this case a Berkovich pyramidal tip has a contact area of *A*_C_ = 24.5*h*^2^, where *h* represents the depth. The initial slope on the unloading branch (stiffness, *S*) is related to the reduced elastic modulus (*E*_r_) by the following relationship:2
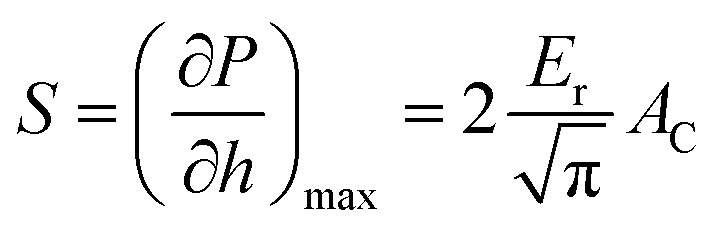
and *E*_r_ is related to elastic modulus *E* with the following equation:^36^3
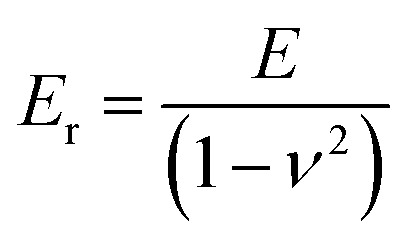
where, *ν* is Poisson's ratio of the samples. In addition, the elastic recovery parameter (ERP) is calculated as follows:4
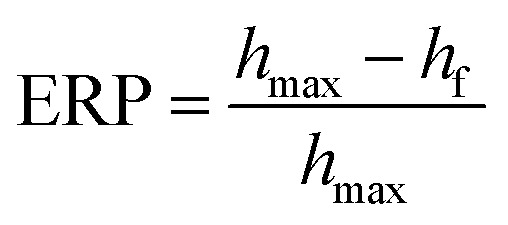
where, *h*_max_ is the maximum indentation depth and *h*_f_ is the non-recovered depth of the indenter inside the sample. The maximum depth applied in each indentation was 22 μm.

### 2.5. Drug loading and release experiments

Drug loading was carried out with 10 mg of PEG particles, where particles were incubated overnight in 1 mL of an aqueous loading solution containing 200 μg mL^−1^ concentration of the drug at room temperature. UV-visible spectrophotometer (Thermo scientific Nanodrop ND100) at 233 nm wavelength was used to measure the amount of drug loaded into the particles. A standard calibration curve for the absorption of the drug in aqueous solution was generated to determine drug concentration in unknown samples. The drug loading efficiency was calculated as follows

5 Drug loading efficiency = (*M*_0_ − *M*_f_)/*M*_0_ × 100

where *M*_0_ represents the initial mass of the drug in the stock solution and *M*_f_ is the final mass of the drug remaining in the solution. To measure the release of the drug from particles, PEG particles in dialysis membrane bag were incubated in 20 mL of PBS, and 0.2 mL samples were taken at 0.5, 1, 2, 4, 12, 24, 48 and 72 hour time points and sample volume was replaced with fresh PBS. Concentrations calculated from drug absorption measurements were used to calculate the mass released at time *t* (*M*_*t*_) as follows:^38^

6 *M*_*t*_ = *C*_*t*_ × *V* + ∑*C*_*t*−1_ × *V*_s_

where *C*_*t*_ is the concentration of drug in the release solution at time *t*, *V* is the total volume of the release solution (12 mL) and *V*_s_ is the volume of the sample taken (0.8 mL). Percent of cumulative drug release, *Q*, was determined as follows:

7 *Q* = (*M*_*t*_/*M*_∞_)100

where *M*_∞_ is the total weight of the drug loaded into particles.

For investigation of drug release from SBS-PEG hybrid gels, drug loaded PEG particles were encapsulated in SBS membrane or crosslinked SBS gels. SBS membrane-PEG and crosslinked SBS-PEG hybrid gels were incubated in 12 mL of PBS, and 0.2 mL samples were taken for 28 days and samples were replaced with fresh PBS. Drug concentrations obtained from absorption measurements were used to calculate the mass of the drug released at specific point in time (eqn (6)) and percent of cumulative amount of the released drug was determined using eqn (7).

### 2.6. Cell survival experiments

Human kidney cells (HEK293, passage number: 7) were cultured at 37 °C with 5% CO_2_ in an incubator and maintained in DMEM supplemented with 1% penicillin/streptomycin, 2% l-glutamine and 10% FBS. 1 × 10^4^ cells per gel were dynamically seeded on SBS and SBS-PEG network samples. Next, samples were placed in the incubator and the CellTiter-Glo luminescent cell viability assay (Promega) was used on days 1, 3 and 5 to characterize the metabolic activity of cells. Percent cell viability for each sample was calculated through normalization with respect to control cells.

## 3. Results and discussions

### 3.1. Synthesis and characterization of PEG particles

**Fig. 2:**
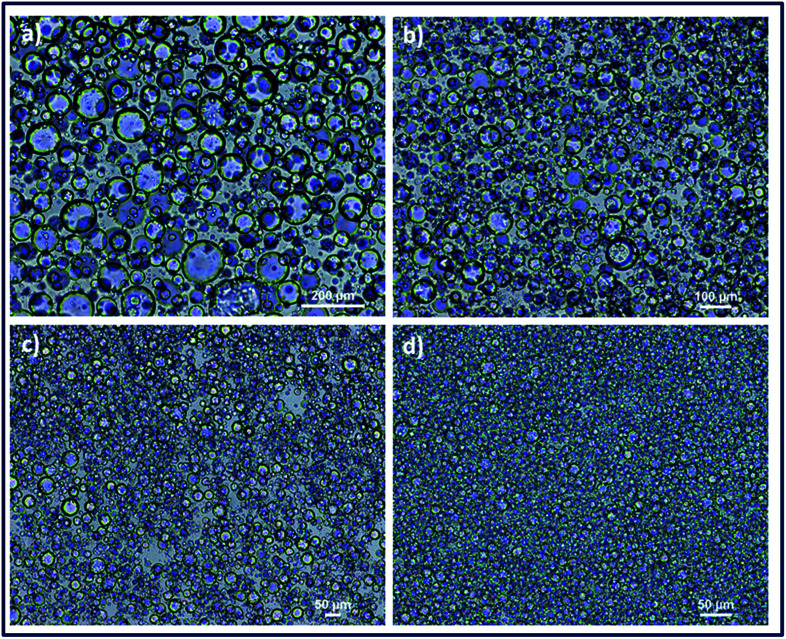
Light microscope images of PEG-cyclohexanone (*φ* = 0.25) water in oil silica nanoparticle (1.5%) stabilized Pickering emulsions prepared by vortex times of (a) 30 s, (b) 60 s, (c) 2 min, (d) 8 min.

**Fig. 3:**
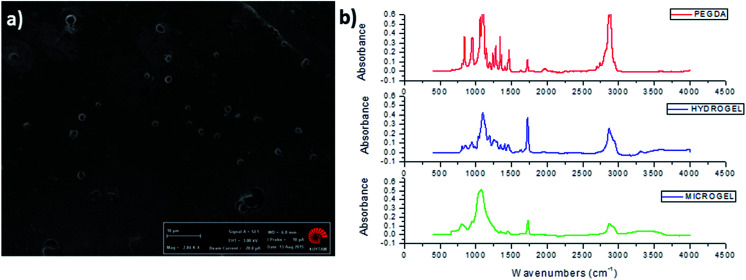
Structural characterization of PEG particles. (a) SEM image of casted PEG particles (scale bar: 10 μm) (b) FTIR spectra of PEGDA, PEG bulk hydrogel and PEG microgel.

Emulsions of PEG-cyclohexanone were prepared by using silica particle stabilized water-in-oil (w/o) Pickering emulsion. PEG prepolymer droplets were dispersed in continuous phase of cyclohexanone with an internal volume fraction (*φ*) of 0.25. Size of the dispersed droplets in particle stabilized PEG-cyclohexane emulsions were adjusted with 1.5% nanoparticle concentration where vortex durations of 30 s, 60 s, 2 min and 8 min were used for optimization experiments. Optical light microscope images of emulsions demonstrated that the smallest size of emulsions could be obtained with the longest vortex time of 8 min (Fig. 2). Fig. 2 shows the effect of vortex duration on the size of droplets in PEG-cyclohexanone Pickering emulsion system. Next, PEG-cyclohexane Pickering emulsions were exposed to UV light to induce photocrosslinking of dispersed PEG prepolymer droplets. Crosslinked particles were retrieved after washing and dried to obtain PEG particles in dry form. Morphology of PEG particles were characterized with SEM. PEG particles for SEM characterization were prepared *via* casting on substrate, and uniform dispersion of particles in SBS is shown in Fig. 3a.

PEG particles were also characterized by FTIR spectroscopy to demonstrate the gelation and crosslinking (Fig. 3b). Weak peak at 1640 cm^−1^ that was assigned to –C=C– stretching occurred in PEGDA monomer, while this peak disappeared after formation of bulk PEG hydrogel and microgel due to consumption of –C=C– double bonds in acrylate groups and formation of a new crosslinked gel network. Strong peaks that appeared between 650 cm^−1^ and 1000 cm^−1^ in PEGDA were attributed to =C–H bending of alkenes, where decrease in the magnitude of these peaks were observed in PEG hydrogel and microgel as a result of consumption of –C=C– double bonds (Fig. 3).

### 3.2. Synthesis and characterization of SBS-PEG hybrid gels

**Fig. 4:**
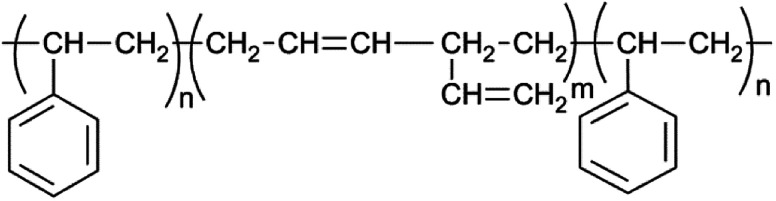
Chemical formula of the SBS rubber.

Crosslinked SBS network was synthesized with the addition of radicalized thiol groups onto the double bonds found in poly(butadiene blocks). Thiol–ene polymerization occurs between the thiol group present in DTT and pendant vinyl double bonds in SBS. Polymerization occurs more efficiently with pendant vinyl double bonds arising from of 1–2 polymerization of butadiene than with the 2-butene double bonds present in the polybutadiene chain backbone (Fig. 4) and vinyl content of SBS largely determines the extent of the thiol–ene crosslinking reactions. The polymer network forms in the elastomeric phase upon exposure to UV through consumption of vinyl groups followed by polymer insolubilization.^1,39^

**Fig. 5:**
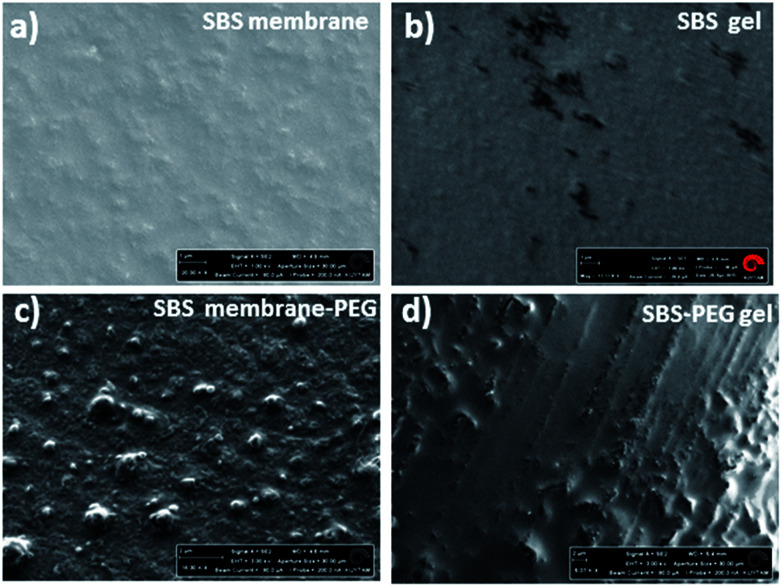
SEM images of (a) SBS membrane (scale bar 1 μm) (b) SBS gel (scale bar 1 μm) (c) SBS membrane-PEG (scale bar 2 μm) and (d) SBS-PEG gel (scale bar 2 μm). SBS membrane-PEG gels were prepared by casting SBS solution after incorporation of PEG particles. SBS gel and SBS-PEG gel were prepared by crosslinking of SBS solution after incorporation of PEG particles. Hybrid gels were dried in vacuum oven overnight before analysis with SEM.

Two different types of hybrid network were prepared: SBS-PEG hybrid gels were prepared by incorporation of crosslinked PEG particles in SBS solution that included TEA, DTT and eosin Y and then network crosslinking of continuous SBS solution was induced under UV-light (365 nm). SBS membrane-PEG samples were prepared as a negative control, where incorporation of PEG particles in SBS solution was followed by casting the PEG particle incorporated SBS polymer solution onto glass substrate surface. SBS gel and SBS membrane samples were also prepared following a similar protocol used for hybrid gels without incorporation of PEG particles. Morphology of SBS membrane and SBS gel with or without PEG particles were observed with SEM (Fig. 5). Surface roughness was evident in both SBS membrane and SBS gel surfaces due to the physically crosslinked nature of SBS with rigid styrene domains (Fig. 5a and b). Uniform distribution of PEG particles was observed both in SBS-PEG samples in SBS membrane and SBS gel (Fig. 5c and d). PEG particles were more clearly distinguished in SBS membrane than in SBS gel (Fig. 5c), probably due to the differences in hydrophilicity between SBS membrane and crosslinked SBS. Crosslinked SBS is more hydrophilic than normal SBS polymer, hence hydrophilic PEG particles could be easily incorporated within crosslinked SBS matrix.

**Fig. 6:**
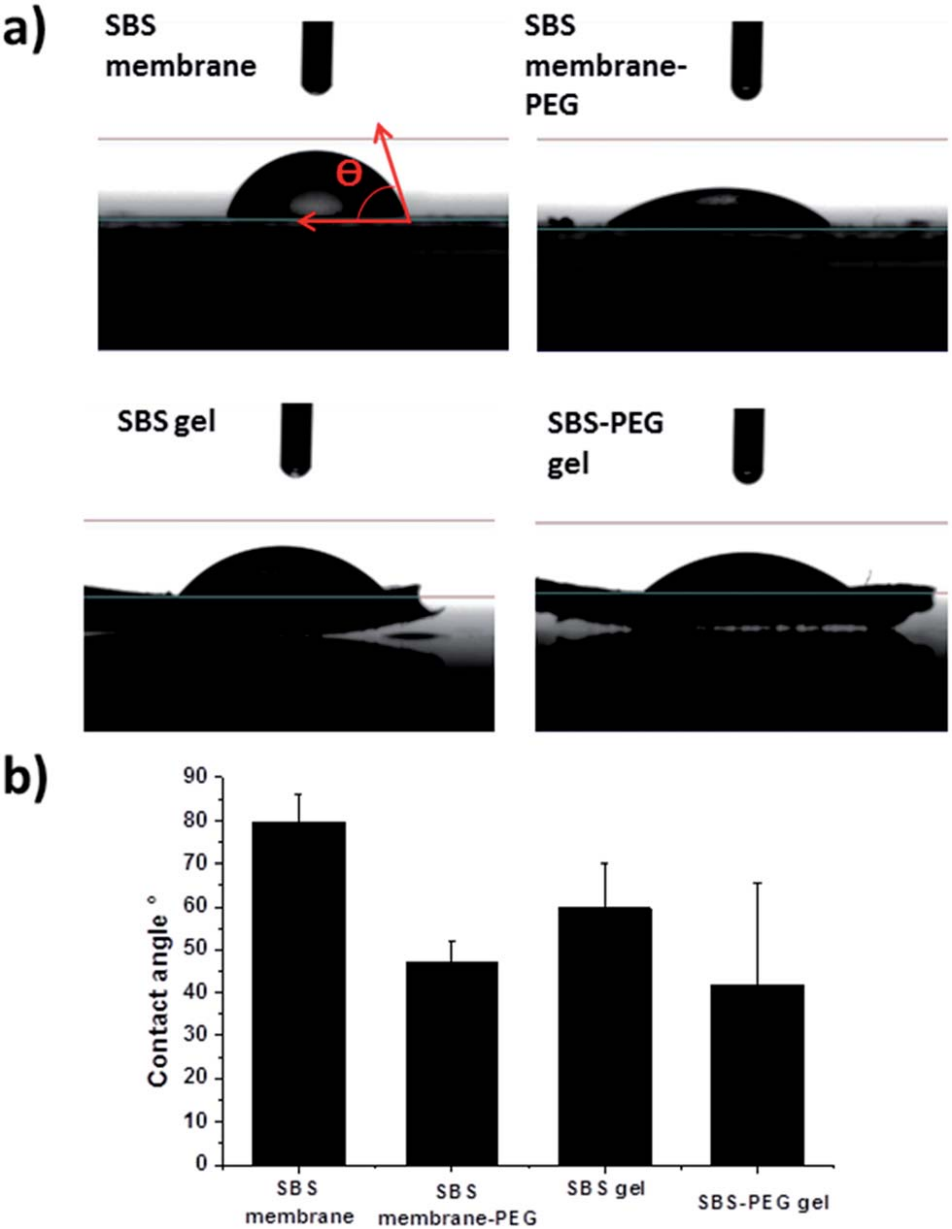
Water contact (a) images and (b) angles (*θ*) of SBS membrane, SBS gel, SBS membrane-PEG and SBS-PEG gel surfaces. 10 μL of deionized, triple distilled water was placed on surfaces for the measurement of contact angles.

Alterations in surface structure and morphology upon PEG hydrogel incorporation within SBS membrane or SBS gels were investigated further with water contact angle measurements. Water contact angles of the droplets on both surfaces were presented in Fig. 6. Water contact angles decreased with crosslinking of SBS and incorporation of PEG particles within SBS membrane or SBS gel matrix (Fig. 6b). This is expected because incorporation of DTT contributes to crosslinking and promotes hydrophilicity in SBS. In addition, incorporation of PEG particles improved hydrophilicity of the hybrid gel further due to hydrophilic nature of PEG hydrogel particles. Further characterization of SBS membrane, SBS-membrane-PEG, SBS gel and SBS-PEG gel were carried out with FTIR and these results have been presented in Fig. S1.†

### 3.3. Nanoindentation tests

The degree of crosslinking of polymers is related to the number of groups that interconnect two materials. Crosslinking density of an elastomer increases with increases in the degree of crosslinking of elastomeric network.^40,41^ In our previous study, we reported increasing crosslinking densities from 2 to 643 mol m^−3^*via* crosslinking of SBS elastomer through photoinitiated thiol–ene reactions and addition of di-thiol linkers within elastomeric network.^3^ Increase of crosslinking degree improves the solvent resistance and mechanical properties.^42^ In addition, increases in the degree of crosslinking promote stiffness of the gel network and slow down the release of molecules from gel network. Here, depth sensing nanoindentation tests were conducted to investigate the mechanical properties of dry SBS gel and SBS-PEG hybrid gels. Characterization of SBS or crosslinked SBS by means of nanoindentation was not reported previously. Nanoindentation is a useful tool that allows direct measurement of the mechanical properties of materials. By indentation of a material to a desired depth followed by retraction of the tip, a load–displacement curve, which is a characteristic of the material being tested, can be obtained. Maximum depth of 22 μm was applied for each indentation. Average elastic modulus and hardness were calculated based on these curves by using Oliver–Pharr method. Reduced elastic modulus and stiffness of the gels were calculated using eqn (2) and (3), respectively. Calculations were performed with a Poisson's ratio of 0.5, based on previous reports about elastic polymers for mechanical characterization.^43,44^ Non-recovered depth, *h*_f_, obtained from load–displacement curves were 4.028 and 3.848 μm for crosslinked SBS gel and SBS gel incorporating PEG microparticles, respectively. ERP of the gels were also calculated based on these values using eqn (4). Average elastic modulus, hardness, stiffness and ERP values are summarized in Table 1.

**Table 1:** Average elastic modulus and average hardness of crosslinked SBS and SBS-PEG gels calculated from load–displacement curves by using Oliver–Pharr method

	Average elastic modulus (*E*) (GPa)	Average hardness (*H*) (GPa)	Average stiffness (*S*) (kN m^−1^)	ERP
SBS gel	0.233	0.080	26.0	0.817
SBS-PEG gel	0.267	0.103	29.7	0.825

**Fig. 7:**
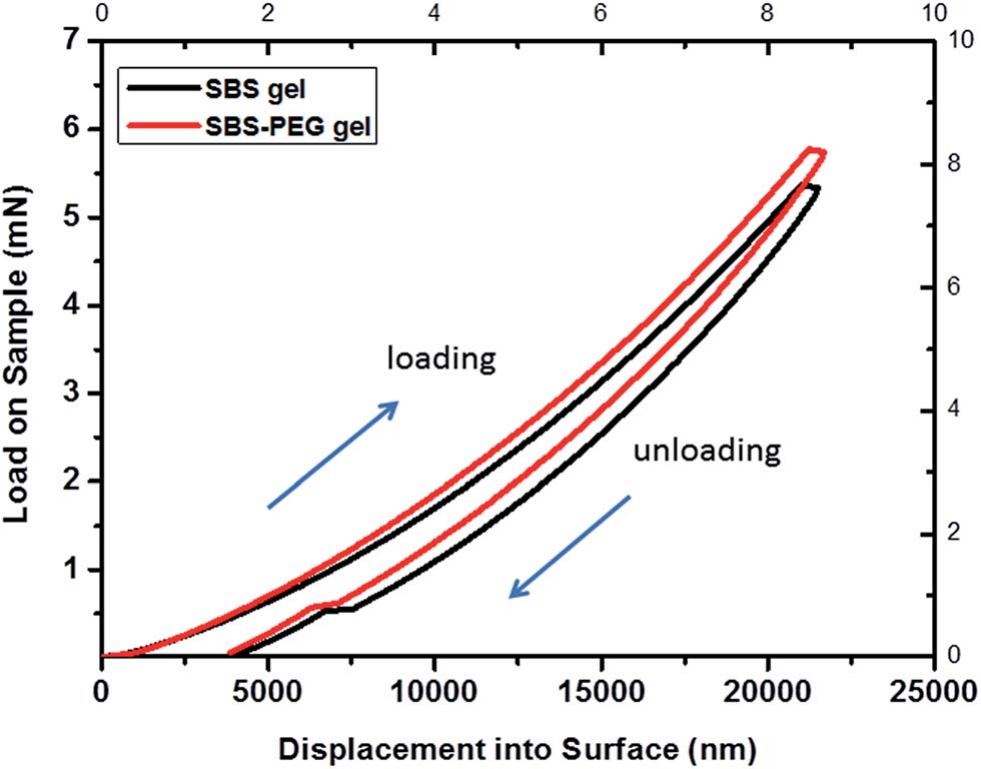
Nanoindentation load–displacement curves for SBS gel and SBS-PEG gel. Maximum indentation depth applied was 22 μm.

The load–displacement curve in Fig. 7 demonstrated that the load measured for maximum depth of indentation was within range of 5–6 mN for dry crosslinked SBS gels and SBS gel incorporating PEG microparticles. Mechanical characterization of hydrogels using nanoindentation is also challenging compared to metals or ceramics, and so there is only limited data available from nanoindentation analysis of gels.^45^ In a previous study, load–displacement curves of epoxy hydrogels were investigated by nanoindentation tests and load range of 1.5–2 μN was applied for a maximum indentation depth of 1.2 μm.^46^ In another study, the load applied for maximum indentation was 2.0–2.5 mN for dry PHEMA hydrogels while it was only 0.5 mN for hydrated ones.^47^ Here, crosslinked SBS gel and SBS gel incorporating PEG microparticles were investigated in dry form and these were stiffer in structure compared to hydrogels investigated in the aforementioned studies. Hence, it is expected to obtain the load within the range of 5–6 mN for crosslinked SBS gel and SBS gel incorporating PEG microparticles samples.

According to the load–displacement profiles measured, a slight increase occurred for each applied load to reach the same indentation with the addition of PEG particles into SBS gel matrix.

Average elastic modulus obtained from these curves were 0.233 and 0.267 GPa for crosslinked SBS gel and SBS gel incorporating PEG microparticles, respectively. It was observed that hardness, stiffness and ERP values, which show the mechanical properties of gels, were slightly higher for SBS gels incorporating PEG microparticles compared to that of crosslinked SBS gels (Table 1). This increase could be explained from the addition of particles into a polymer system, as incorporation of nanoparticles or nanotubes in materials also resulted in enhanced ultimate tensile strength in previous reports.^48,49^

### 3.4. Drug release from PEG particles and SBS-PEG gels

**Fig. 8:**
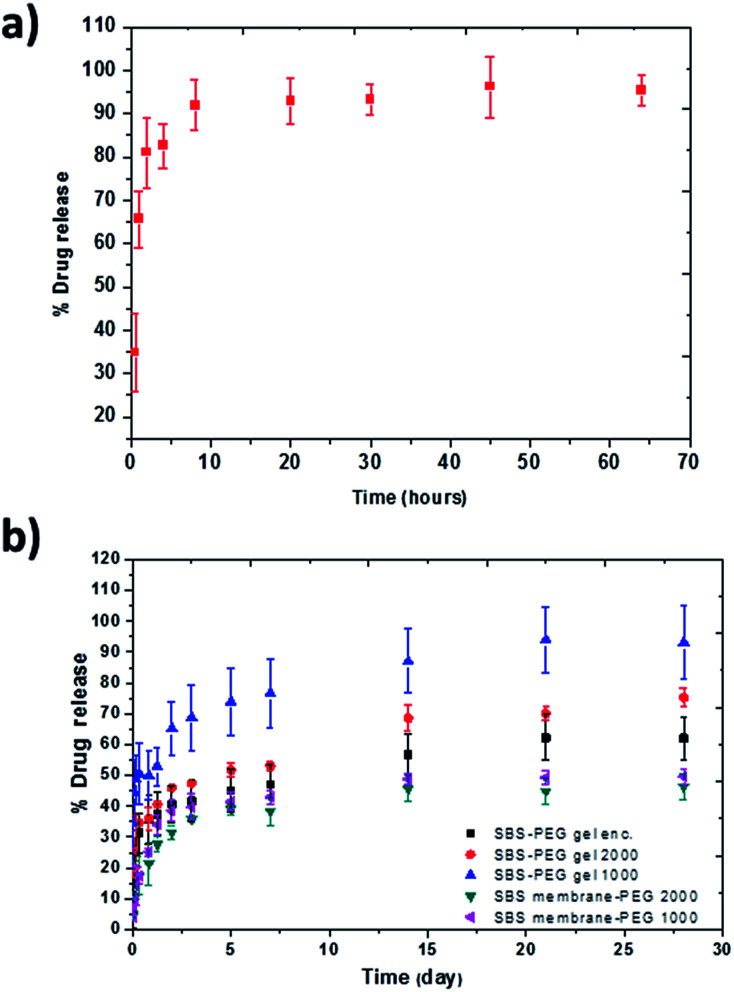
Cumulative percent release of drug from (a) PEG particles and (b) SBS membranes or SBS-PEG gels incorporating PEG microparticles.

PEG hydrogel particles were incubated in drug solution and percent loading into PEG particles was calculated as 85 ± 4.2% using eqn (5). For release experiments, PEG particles were incubated in PBS at physiological pH (7.4) for 72 h and 0.2 mL samples were taken from each set at 0.5, 1, 2, 4, 12, 24, 48 and 72 hour time points and the same volume was replaced with fresh PBS. Concentration of the drug in samples at different time points were measured by UV-visible spectrophotometer at 238 nm wavelength based a calibration curve that we established with known concentrations of the drug. Time dependent mass and percent drug release were calculated based on these measurements with eqn (6) and (7). Percent cumulative drug release from PEG particles is presented in Fig. 8a. Maximum cumulative release of 95.5 ± 3.5% was obtained at 72 h, where cumulative release of 91.9 ± 5.8% was measured from PEG particles during the first 8 hours in PBS which was characterized as burst release (Fig. 8a).

Next, PEG particles were incubated in 1000 and 2000 μg mL^−1^ concentrations of drug where percent drug loading were measured as 61.1 ± 11.0% and 57.7 ± 7.0%, respectively. After drying, drug loaded PEG particles were encapsulated in either SBS membranes or crosslinked SBS gels with 0.5 mg particles in 300 μL SBS solution to obtain SBS membrane-PEG or SBS gel-PEG system. P2X7R antagonist drug was also encapsulated in PEG particles as an additional group, and these particles were then incorporated within SBS gel network in subsequent experiments. Amount of drug encapsulated in SBS solutions were calculated based on the amount of drug incorporated within PEG particles.

SBS-PEG gels were incubated in PBS for 28 days and drug release from SBS-PEG samples were measured *via* UV-visible spectrophotometer absorbance at 233 nm. Percent drug release from altered networks can be observed in Fig. 8b.

Continuous drug release was observed up to 28 days for all samples prepared with SBS membrane or SBS gels. Drug molecules loaded in PEG particles diffuse out from PEG particles through hydrophobic SBS gel and from SBS gel towards PBS environment subsequently. The possible drug release mechanism for release of P2X7R from SBS-PEG gels are illustrated in Fig. S2 (ESI†). This sustained release profile can be attributed to the differences in hydrophobic–hydrophilic nature of SBS-PEG and amphiphilic property of the drug, which might result in localization of the drug at the SBS-PEG particle interface. This localization might also limit drug diffusion from SBS matrix towards PBS. The second reason for sustained release could be due to low mesh size of SBS network which would slow down the diffusion of the drug. Fig. 8b demonstrated that crosslinked SBS system allowed for higher percent drug release compared to that of casted SBS membrane due to the porous gel network structure. This crosslinked network enhanced the diffusion of drug and less amount of drug was entrapped in this system compared to that of SBS membrane. The more hydrophilic structure obtained with SBS crosslinking also contributed to the loading of the drug into the gel and enhanced diffusion of drug towards the gel surface. Higher drug release percentages were measured in drug loaded systems prepared with SBS gel-PEG1000 and SBS gel-PEG2000 compared to that of SBS gels incorporating PEG microparticle encapsulated system, where drug was encapsulated within PEG particles (black squares, Fig. 8b). This higher release with drug loaded systems were probably due to both loading of the drug inside PEG particles and adsorption onto the surface of PEG particles, which promoted the diffusion of the drug easily through SBS matrix and led to higher percent of drug release. Another significant observation was the higher amount of drug release observed in SBS gels incorporating PEG microparticles loaded with 1000 μg mL^−1^ drug solution compared to the group that was suspended in 2000 μg mL^−1^ drug solution. This could be due to intense drug–drug interactions and aggregations at high concentrations which compromised drug diffusion through crosslinked SBS network (Fig. 8b). Various studies focused on extended release profiles for various drugs through different networks such as composite polymers, hydrogels and reported that matrix systems that included nanoparticles were promising for this purpose.^13,14,50^ For example, in one study, PLGA nanoparticles were incorporated within chitosan scaffolds to control BSA delivery and continuous release of BSA up to 25 days was observed.^51^

### 3.5. Biocompatibility tests of SBS and SBS-PEG gels

**Fig. 9:**
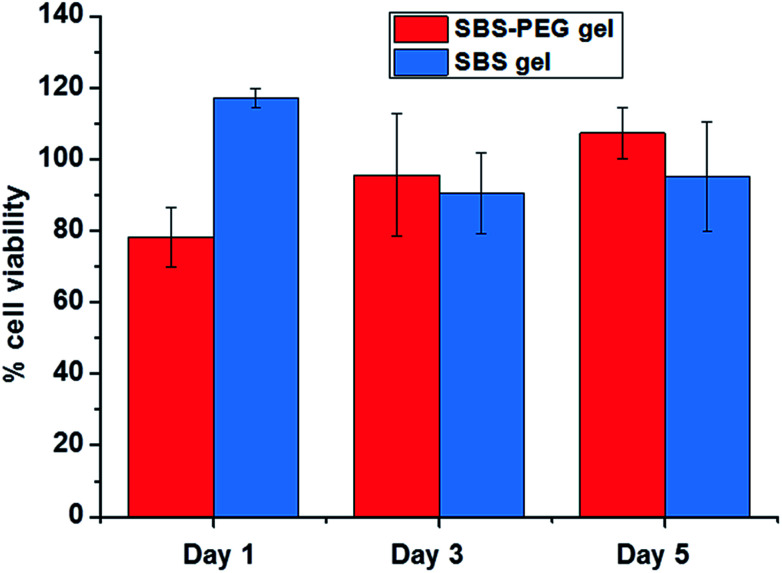
Percent cell viability of SBS gel and SBS-PEG gels.

To investigate biocompatibility of SBS gels and SBS-PEG gels incorporating PEG microparticles *in vitro*, viability of human embryonic kidney (HEK 293) on both surfaces were measured. Percent cell viability results demonstrated that crosslinked SBS and SBS gels incorporating PEG microparticles did not compromise cell proliferation and growth (Fig. 9). All samples had high cell survival of above 80% compared to control group.

Adhesion experiments of SBS and SBS-PEG hybrid gel on human skin was also tested. Adhesion of the hybrid gels onto the human skin was observed in these tests. Gels were not detached from the skin with upside down movements of the arm. No irritation, itching or other reaction was observed due to application of the gels. An image and a video of this test are presented in ESI (Fig. S3†).

## 4. Conclusions

We developed a novel crosslinked SBS hybrid gels incorporating PEG microparticle platform for sustained delivery of an anti-rheumatoid arthritis drug. PEG hydrogel particles were incorporated with SBS prepolymer which was subsequently crosslinked through photopolymerization to form a hybrid SBS polymer network. Gels were characterized through SEM, water contact angle measurements and mechanical properties were investigated with nanoindentation experiments. We measured the release of an anti-rheumatoid arthritis drug from these SBS-PEG gels and observed sustained drug release up to 28 days for the first time. We demonstrated the effect of hydrophilic–hydrophobic nature and crosslinking of SBS on drug release properties. Cell survival analysis with HEK293 revealed that the composites prepared here are non-toxic and biocompatible. These results suggested the potential of SBS-PEG gels for sustained drug delivery applications.

The approach presented here with crosslinked, amphiphilic and elastic SBS gel systems is not only promising for extended release of P2X7R antagonist but could also allow for incorporation of different molecules for simultaneous/sequential therapeutic molecule delivery.

## Conflicts of interest

There are no conflicts to declare.

## Nomenclature

SBS membrane: SBS solution casted on substrate
SBS membrane-PEG: PEG particles incorporated within SBS membrane
SBS gel: Cross-linked SBS
SBS-PEG gel: PEG particles incorporated within cross-linked SBS hybrid gel

## Supplementary Material

RA-008-C8RA01460D-s001

RA-008-C8RA01460D-s002

RA-008-C8RA01460D-s003
