# Supplementary material for: P2X7 receptor antagonist delivery vehicle based on photocrosslinked amphiphilic hybrid gels
Source: RSC Adv. 2018 May 18;8(33):18216–26. doi: 10.1039/c8ra01460d (PMC9080578; doi:10.1039/c8ra01460d)
Supplement: RA-008-C8RA01460D-s003 [file RA-008-C8RA01460D-s003.pdf]

Electronic Supplementary Material (ESI) for RSC Advances.
This journal is © The Royal Society of Chemistry 2018

P2X7 Receptor Antagonist delivery vehicle based on photocrosslinked amphiphilic hybrid gels

Derya Aydin and Seda Kizilel *

Department of Chemical and Biological Engineering, Koc University, Sariyer, Istanbul, Turkey, 34450

*E-mail: skizilel@ku.edu.tr
Fax:+90-212-338-1548

SUPPLEMENTARY INFORMATION

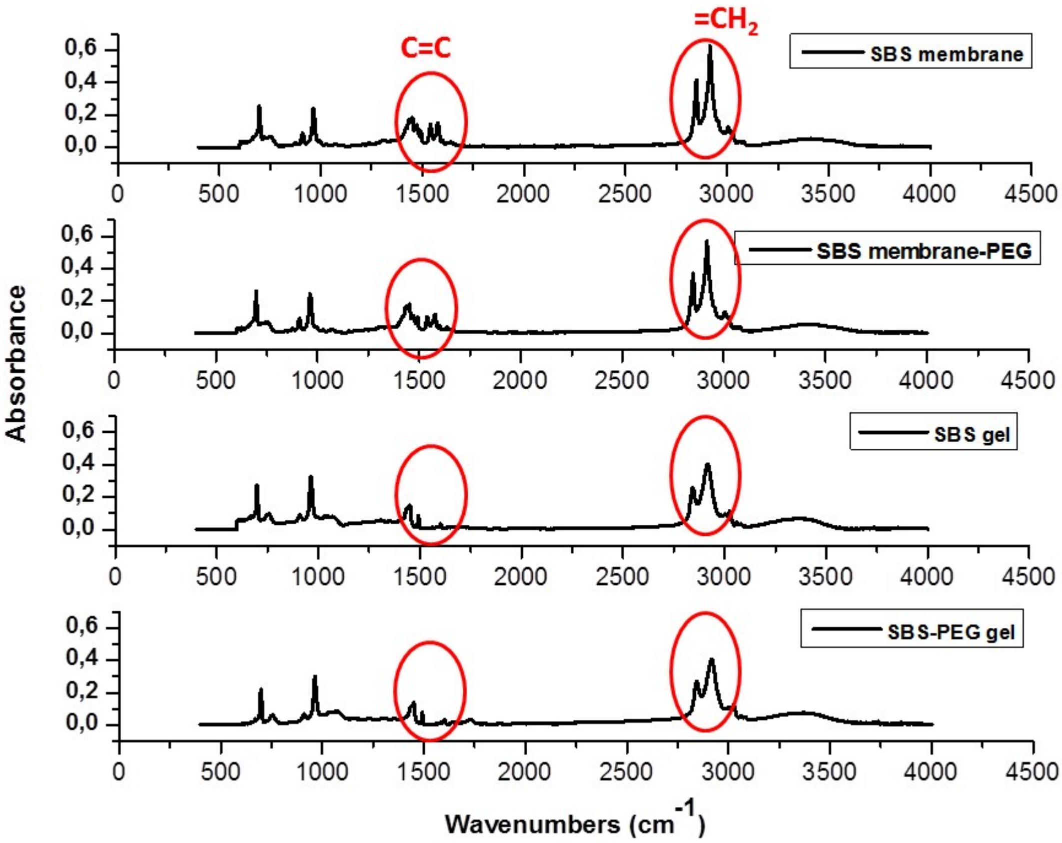

Figure S1. FTIR spectra of SBS membrane, SBS membrane-PEG, SBS gel and SBS-PEG gel

Figure S1 demonstrates the peaks related to the asymmetrical (at 2920 cm^{-1}) and stretching (at 3006 cm^{-1}) vibrations of $=CH_2$ groups in pendant vinyl double bonds of butadiene. Decreases in the peaks at 2920 cm^{-1} were observed in SBS gel and SBS-PEG gel groups compared to

that of SBS membrane and SBS membrane-PEG groups. This result could be attributed to the saturation of vinyl groups in the SBS structure in crosslinked SBS and SBS-PEG gels samples.

The peak at 1639 cm^{-1} can be assigned to stretching vibrations of (C=C) in vinyl-vinyl-polybutadiene groups. Decreases in this peak in SBS gel and SBS-PEG gels could be attributed to the consumption of these double bonds in SBS upon crosslinking. The peaks at the 1580 cm^{-1} and 1602 cm^{-1} were assigned to stretching vibrations of the carbons in the aromatic rings where these peaks disappeared in crosslinked SBS and SBS-PEG gel networks as a result of the formation of a stable network. This analysis confirms the successful cross-linking of SBS polymer both in SBS and SBS-PEG gels.

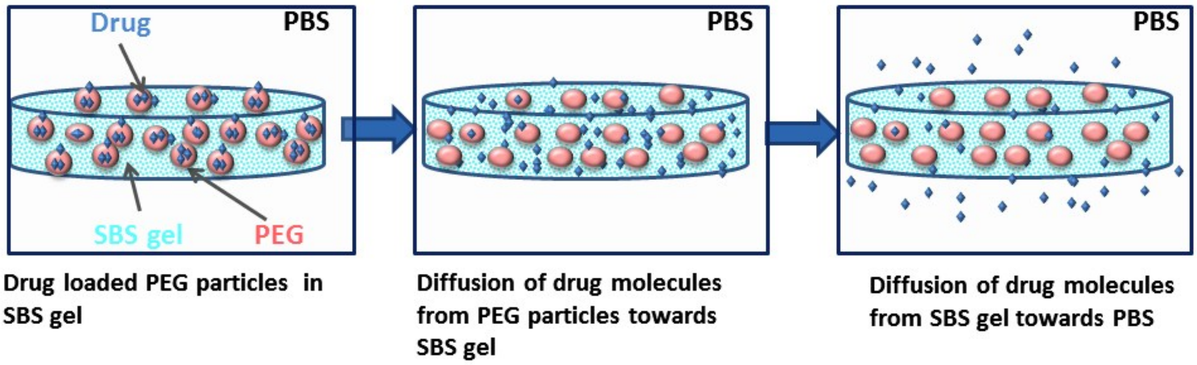

Figure S2. Possible diffusion process/mechanism for the release of the P2X7 from SBS-PEG gels towards PBS.

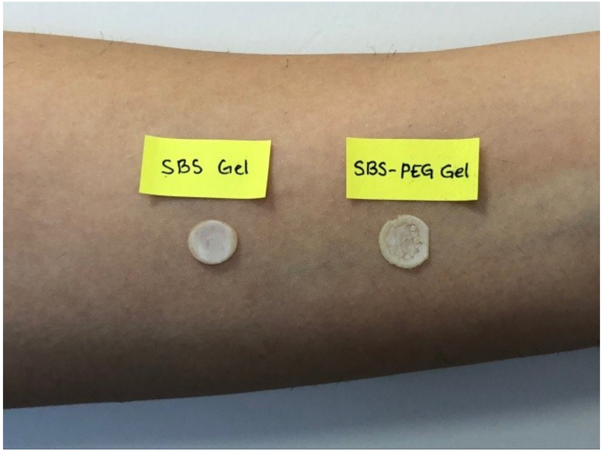

Video 1.mp4

Figure S3. Adhesion and biocompatibility experiment of SBS gel and SBS-PEG gel on human skin.
